# Management of Pheochromocytomas and Paragangliomas: A Case-Based Review of Clinical Aspects and Perspectives

**DOI:** 10.3390/jcm11092591

**Published:** 2022-05-05

**Authors:** Bartosz Kamil Sobocki, Adrian Perdyan, Olga Szot, Jacek Rutkowski

**Affiliations:** 1 Student Scientific Circle of Oncology and Radiotherapy, Medical University of Gdansk, 80-214 Gdansk, Poland; olga.szot@gumed.edu.pl; 2 International Research Agenda 3P Medicine Laboratory, Medical University of Gdansk, 80-210 Gdansk, Poland; perdyan.adrian@gumed.edu.pl; 3 Department of Oncology and Radiotherapy, Medical University of Gdansk, 80-214 Gdansk, Poland

**Keywords:** pheochromocytoma, paraganglioma, neuroendocrine tumors, radiology, nuclear medicine

## Abstract

Paraganglioma and pheochromocytoma are rare medical conditions. Thus, there are still a small number of studies, clinical trials, and evidence-based data in this field. This makes clinical decisions more difficult. In this study, we present a case report enriched with a short review of available essential clinical data, indicating the need for constant metoxycatecholamine level observation and a proper diagnostic imaging approach, especially in terms of ongoing pandemics. Our research also provides a summary of the molecular background of these diseases, indicating their future role in clinical management. We analyzed the ClinicalTrials.gov dataset in order to show future perspectives. In this paper, the use of the PET-CT before MRI or CT is proposed in specific cases during diagnosis processes contrary to the guidelines. PET-CT may be as effective as standard procedures and may provide a faster diagnosis, which is important in periods with more difficult access to health care, such as during the COVID-19 pandemic.

## 1. Introduction

Pheochromocytoma (PCh) is a tumor located in an adrenal gland, whereas paraganglioma (PGL) is an extra-adrenal tumor that is commonly formed near blood vessels and nerves [1]. The nomenclature of these rare neuroendocrine tumors (NETs) depends on primary location, as they lack relevant differences in their histology [1,2]. Both PCh and PGL have similar prognoses—their five-year overall survival (OS) is evaluated to be approximately 95% in nonmetastatic disease and 34–60% in recurrent or metastatic neoplasms [3]. At the same time, the incidence rate of PCh and PGL is rising [4,5,6]. This may be associated with better access to cross-sectional imaging; hence, a 28.1% greater detection rate of PCh and PGL was observed between the periods of 1995–2004 and 2005–2016 [7,8].

The main risk factors include genetic predisposition, age between 30 and 50, race/ethnicity (the tumors are less common in African Americans), and Carney triad (a nonhereditary multitumoral syndrome that affects young women) [9,10]. Hypoxia may also play a crucial role in PCh and PGL tumorigenesis, and every 500 m increase in elevation contributes to an 0.31/100,000 increase in the prevalence of these tumors.

**Figure 1:**
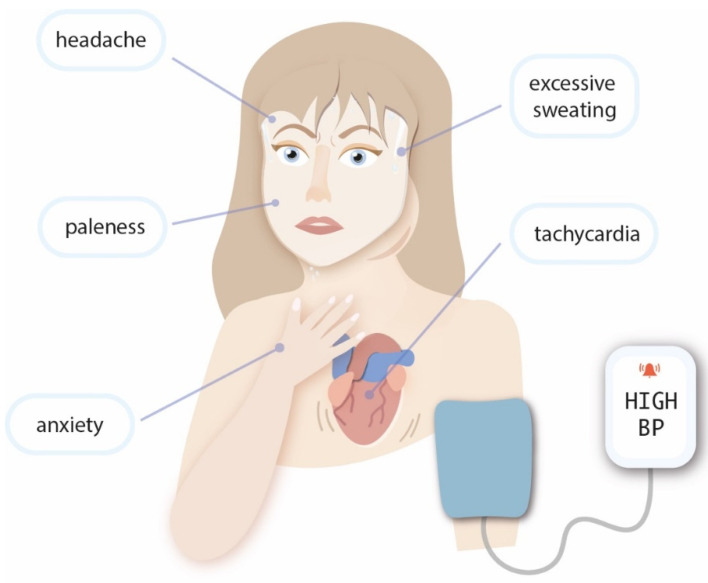
The clinical symptoms of pheochromocytoma and paraganglioma.

As PGL may present similar symptoms to PCh when secreting catecholamines, the differentiation of the tumors can be challenging. However, if it is hormonally inactive, it might be asymptomatic, even in advanced stages. The most common symptoms are tachycardia, hypertension, headache, paleness, excessive sweating, and anxiety (Figure 1) [1]. The most common location of catecholamine secreting PGL is the abdomen and pelvis [1]. Head-and-neck PGL tumors are rarely hormonally active; thus, symptoms are associated with their location [11]. PGL may cause a mass effect in the neck, and if more advanced, it may lead to dysphonia, dysphagia, or neck pain [11]. Moreover, glomus tympanicum tumor and temporal paragangliomas manifest through hearing impairment, tinnitus, and balance disturbance [11]. Glomus jugulare, glomus vagale, and carotid body tumors can present with cranial nerve (IX-XII; X-XII; X, XII) palsies, respectively [11]. The purpose of this review was to provide a relevant summary of PCh and PGL management, especially in the clinical setting, and to discuss the current guidelines in the context of the presented case report.

## 2. Case Report

In January 2018, a 38-year-old woman was admitted to the surgical department for open resection of a solid-cystic lesion (94 × 80 × 70 mm), discovered in regular ultrasound examination and further revealed in computed tomography (CT). The tumor was localized in the tail of the pancreas with potential involvement of surrounding tissues, including the adrenal glands. CA125, CEA, and Ca19.9 were within normal ranges. On her first admission to the clinic (a day before planned surgery), she presented with heart palpitations, headache, blood hypertension, and petulance. The patient’s only comorbidity was Hashimoto’s disease (HD). Her family history was positive for HD but negative for cancer. The tumor was completely removed, along with its capsule and the whole left adrenal gland. The remaining part of the pancreas was surgically released and secured. The histopathological examination confirmed the PCh diagnosis. The tumor was characterized by the presence of highly atypical cells, division figures (5/50 high power fields), positive chromogranin A (CgA) and synaptophysin (Syn), and partially positive S-100 protein, as well as a Ki67 index of 1%.

**Figure 2:**
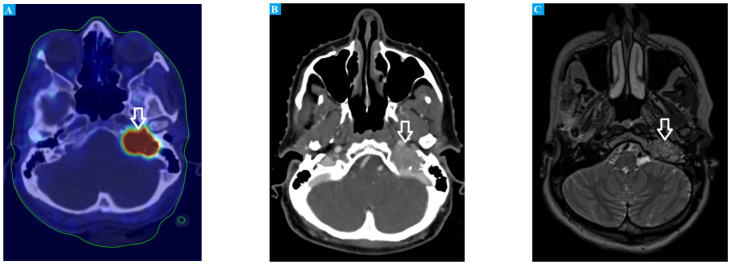
(**A**) combined ^68^GA-DOTATE PET and CT images showing a large, highly metabolically active mass in the skull; (**B**) CT scan showing a polycystic tumor compressing the left cerebellar tonsil; (**C**) combined MR and CT scan showing infiltration of the left sublingual nerve extending to the left internal carotid artery canal and obstructing the left internal jugular vein at the inferior bulb region.

After the surgery, our patient was initially followed up by the surgical outpatient clinic. Unfortunately, the next follow-up at an endocrinological outpatient clinic was canceled, due to the lack of emerging symptoms and prolonged waiting time for an endocrinological consultation (which, in Poland, is 1–2 years) [12]. In January 2020, due to the appearance of similar symptoms as those before the surgery (such as heart palpitations and anxiety), the patient ended up in a private health care clinic. After endocrinological consultation and laboratory tests, further care was recommended in an oncological outpatient clinic. At that time, levels of metoxycatecholamines in the patient’s 24 h urine test were as follows: metanephrine—550 µg/24 h (reference limit: 74–297 µg/24 h), 3-methoxytyramine—1047 µg/24 h (reference limit: 94–400 µg/24 h), and normetanephrine—6902 µg/24 h (reference limit: 105–354 µg/24 h). These results, together with the patient’s clinical symptoms, raised suspicion of recurrence. Somatostatin receptor scintigraphy and computed tomography (SPECT) focused on the head, chest, abdomen, and pelvis were performed and showed no signs of local or distant relapse. Moreover, positron emission tomography (PET) was ordered in search of a secondary lesion. However, due to the COVID-19 pandemic and national lockdown in Poland, our patient refused to perform PET as soon as possible; hence, the procedure was postponed until August 2020. In June 2020, the patient performed another full-body Tc-99-octreotide somatostatin receptor scintigraphy and CT, but the radiological picture was stationary (no sign of relapse). Finally, ^68^GA-DOTATE PET (combining somatostatin analog tyrosine-3 octreotate with the PET tracer gallium Ga68) was performed, and the new highly metabolically active lesion of the neck and the skull base was detected (standardized uptake value: 15.1) (Figure 2A,B). Further, the patient was admitted to the Department of Oncology and Radiotherapy in September 2020. In order to precisely assess local advancement before the planned treatment, a magnetic resonance (MR) of the head and neck with intravenous contrast was performed. A polycystic tumor putting pressure on the left tonsil of the cerebellum (without infiltration or edema) was observed. The tumor infiltrated the left sublingual nerve area and reached the left internal carotid artery canal. Moreover, it closed the left internal jugular vein in the inferior bulb region (Figure 2C). Based on clinical and radiological images, unresectable PGL (Fishers’ D1) was diagnosed. Hence, from October to November 2020, radical volumetric arc radiotherapy (RT) was undertaken within the area of the tumor and the base of the skull, with adequate margins (Varian, TrueBeam linac; 6 MV photons; D = 54 Gy/27 fr). The treatment tolerance was good; the patient suffered from grade 1 mucositis (Common Toxicity Criteria version 5) and pain for 4 weeks after RT. Four months after RT, in March 2021, significant biochemical regression in the 24 h urine test was observed (metanephrine—88.9 µg/24 h; 3-methoxytyramine—356.5 µg/24 h; normetanephrine—1858.2 µg/24 h), together with a reduction in the initial symptoms (palpitation, petulance). Until now, the patient’s general condition is good and is monitored in our clinic every 3 months. The levels of metoxycatecholamines are slowly decreasing, and in August 2021, the lab tests revealed the following results: metanephrine—65.1 µg/24 h, 3-methoxytyramine—224.8 µg/24 h, and normetanephrine—1180.5 µg/24 h. We summarized the changes in clinical findings in Table 1.

## 3. Genetic Background

Genetics plays a major role in the characteristics of PCh and PGL and has an impact on tumor location, clinical symptoms, hormonal activity, and characteristic imaging phenotype [13,14]. Currently, there are numerous PCh- and PGL-associated genetic syndromes reported in the literature such as MEN-2 (germline mutation in proto-oncogene RET), von Hippel–Lindau (mutation in suppressor gene VHL), neurofibromatosis type 1 (mutation in suppressor gene NF1), PGL syndromes 1–5 (mutations in succinate dehydrogenase subunits SDHD, SDHAF2, SDHC, SDHB, and SDHA, respectively), and hereditary syndromes (mutation in transmembrane protein 127 and MYC-associated factor X genes) [1]. Jochmanova et al. divided all these mutations in PCh and PGL into three clusters according to functional phenotypes, i.e., pseudohypoxic signaling, kinase signaling, and wingless and Int-1 (Wnt) signaling clusters [14]. Interestingly, the expressions of genes belonging to each group had similar clinical images. We divided the genes into three groups and summarized all clinically important data found in the literature in Table 2.

## 4. Diagnosis

### 4.1. Measurement of Metanephrines

The diagnosis of PGL tumors should be based on a plasma or urinary metanephrines analysis, as it is a quick and noninvasive approach. The patients can be divided into two groups based on the risk of PCh or PGL development, i.e., a high-risk group (harboring paternal mutation) and a low-risk group (those who inherited a pathogenic variant influencing the maternal gene). Although, in general, there is no significant difference in the diagnostic value between plasma and urine, plasma is the preferred material among high-risk patients, since it shows higher specificity and sensitivity in comparison to urine analysis [32]. In dopamine-secreting PGL, plasma 3-methoxytyramine level should also be measured, as it is a specific marker of this type of tumor [32]. We summarized the information about diagnosis with metanephrine detection from Szosland et al. study and updated the recommended method with the results of Därr et al., as shown in Table 3 [33,34].

Usually, in clinical practice, a 24 h urine test for deconjugated metanephrines (though it gives numerous false-positive results) and measurement of free metanephrines in plasma are performed [32]. Därr et al. reported that the most accurate method of measurement for metanephrines is high-performance liquid chromatography (HPLC) with both coulometric/electrochemical or tandem mass-spectrometric methods, as it provides a sensitivity of 94% and specificity of 93% [34]. Interestingly, a supine position during blood sample collection provided significantly higher sensitivity in comparison to a seated position (not observed for 24 h urine test), whereas specificity was higher in a group with samples collected in the sitting position than in a group with performed 24 h urine test (without relevance of position during sample collection) [34]. To summarize, the best method seems to be the measurement of plasma concentration collected in the supine position. The level of catecholamines can be affected by different drugs, e.g., tricyclic antidepressants, serotonin, norepinephrine reuptake inhibitors, and levodopa. When the increase in metanephrines level is less than fourfold above the upper reference limit, it is beneficial to confirm the diagnosis with a clonidine suppression test, which showed a sensitivity of 100% and specificity of 96% [35].

### 4.2. Imaging

Regarding imaging methods, ultrasonography may play an essential role as the preliminary tool that detects up to 80–90% of PGL tumors [35]. However, despite its high sensitivity, it provides low specificity, which makes it impossible to profile the tumor and plan the proper treatment. According to the Endocrine Society Clinical Practice Guidelines, 2014 CT ought to be performed on the majority of PGL patients to provide the highest detection rate and images that are appropriate for treatment planning. On the other hand, in the case of the skull base and neck PGL, magnetic resonance imaging (MRI) is recommended. This statement is also supported by the Working Group of the European Society of Hypertension guidelines, which confirm that an MRI is a first-line imaging method for head-and-neck tumors, whereas CT is recommended for tumors located in the rest of the body. When disseminated PGL is suspected, it is advised to use 123I-metaiodobenzylguanidine (MIBG) scintigraphy. However, 18 F-FDG PET/CT should be performed in patients with certain types of metastatic PCh and PGL [36]. In the 2014 guidelines, 123I- MIBG was recommended, whereas, in 2020, its value was considered limited. Nowadays, 18F-FDOPA PET and 68Ga-DOTA-somatostatin analog (SSA) are the most preferable techniques [32,36]. Another available method is somatostatin receptor scintigraphy, known as octreotide scan (111In-pentetreotide). However, due to high cost, high radiation exposure, and lower sensitivity and specificity in comparison with other methods, its use is rather limited [37].

### 4.3. Clinical Presentation Associated with Genetic Background

In general, clinical presentations of PCh and PGL are comparable and depend on hormonal activity and primary localization. However, the presence of each or both tumor types may be a part of multiple hereditary genetic syndromes, which makes diagnosis easier. The most frequent mutations are SDHB—10%, SDHD—9%, VHL—7%, RET—6%, and NF1—5–7% (Table 2) [24,25,26,27,28,29,30,31]. Familial PGL (FPGL) is divided into five subtypes, with impaired tumor suppressor function from which FPGL-4 and FPGL-1 are most frequent. PGL tumors are localized in the abdomen, pelvis, mediastinum, and head-and-neck region. In the second type of von Hipple–Lindau syndrome, PCh is present alongside hemangioblastomas in the retina or central nervous system, clear-cell renal-cell carcinoma, endocrine neoplasia, and pancreatic cancer in different combinations. Proto-oncogene mutation in RET gene is associated with three subtypes of multiple endocrine neoplasia 2 (MEN2) syndromes. In both MEN2A and MEN2B subtypes, PCh may occur with medullary thyroid carcinoma and hyperthyroidism. Additionally, amyloidosis of the skin is present in MEN2A, whereas multiple mucosal neuromas and Marfan-like syndrome are common for MEN2B syndrome. Finally, in NF-1 cafe-au-lait macules, neurofibromas around the head and neck, optic glioma, lish nodules, osseous lesion, and PCh can be observed frequently [16].

### 4.4. Histopathology

Histologically, PCh and PGL are very similar and often indistinguishable. However, some minor differences in cellular structure can be highlighted. PCh cells are characterized by higher nuclear atypia and cytoplasmic basophilia in comparison to eosinophilic PGL cells. Frequently use immunohistochemistry markers are neuron-specific enolase (NSE), S-100 protein, Syn, CgA, cytokeratin, n (CK), and vimentin. Nonetheless, their utility is questionable. S-100 protein may be used to identify Zellballen nests; however, its occurrence was reported in both PCh and PGL [38,39]. Furthermore, in PGL, all the above markers were tested to distinguish benign and malignant lesions. Among others, CK and vimentin seemed to be more frequently expressed in benign tumors (22.2% versus 5.3% and 76.9% versus 12.5%, respectively) [39]. With recent advances in targeted sequencing, the role of immunohistochemistry staining seems to be outdated. Currently, genetic profiling of these tumors with the new generation-sequencing (NGS) method should be performed, as it provides detection of all mutations at the same time, with higher specificity and sensitivity [38].

### 4.5. Differential Diagnosis

PCh and PGL—despite their molecular subtypes—need to be distinguished from other tumors such as subclinical PCh, lipid-poor adenoma, and adrenocortical carcinoma. The first refers to a disease that is not advanced enough to be present with severe clinical symptoms; however, subtle hormonal activity can be identified [40]. Adenomas are benign tumors without the need for special management when detected. To distinguish them from PCh with high specificity, a biphasic, contrast-enhanced CT scan should be performed. PCh, when compared with lipid-poor adenomas, is larger, shows cystic degeneration, and presents higher values in unenhanced, arterial, and venous phases, while the enhancement ratio in arterial and venous phases is lower [41]. Finally, the enhancement loss between PCh and adrenocortical carcinoma is similar; thus, clinical–non-radiological features are key to distinguishing them properly [42]. Most adrenocortical malignant tumors are diagnosed with Cushing’s syndrome and signs of virilization. Additionally, they tend to be much more aggressive, with rapidly increasing clinical symptoms [43,44].

## 5. Treatment

### 5.1. Local Treatment of Early Disease

The first step in catecholamine secreting PCh and PGL management is the administration of α-adrenergic blockers to minimize adverse cardiovascular effects before surgery (nonselective α-blocker phenoxybenzamine is the drug of choice) [45]. For laparoscopic resection of PCh, there is no significant difference in intraoperative hemodynamics, complication rates, and the length of stay in a hospital between groups treated with nonselective and selective α-blockers. However, patients with α-1 selective drugs have a higher risk of transient hypertension during surgery and a greater need for postoperative support [46,47]. To avoid reactive tachycardia caused by α-blockers, the use of β-blockers is recommended (except labetalol) after administering the α-blocker. Prolonged oversecretion of catecholamines reduces blood volume; hence, it is advised to raise sodium and fluid intake perioperatively [36]. The minimally invasive laparoscopic approach is preferable in the surgical treatment of unilateral PCh (≤6 cm) and PGL, with no infiltration of adjacent tissues or metastasis. In cases of bilateral PCh (e.g., in MEN2, VHL), cortical sparing adrenalectomy of one of the glands should be performed [48]. Open surgery should be performed in the case of PCh > 6 cm and PGL located near the aortocaval region [49]. Cervical and intracranial PGL may require more complex techniques due to unfavorable location [50]; therefore, surgery remains the gold standard, and available data show that only complete resection can be potentially curative. Unfortunately, this procedure may be complicated and burdened with a high risk of complications. Embolization may be used as a preoperative therapy to reduce the size of the tumor and minimize the bleeding during an operation—it is indicated in large PGL and PGL of the jugular vein and should be performed 2–3 days before the surgery [51]. If there are technical or medical contraindications for surgery, radiotherapy remains the standard of care, with high local control. Stereotactic radiosurgery is recommended for skull-based tumors smaller than 3 cm [32]. For larger lesions, fractionated and highly conformal techniques are preferable [52]. If the resection was partial, radiotherapy may also be used as a postoperative therapy [53].

### 5.2. Treatment of Metastatic or Relapsed Disease

Unfortunately, approximately 10% of the patients develop metastases, and 6.5–16.5% experience local relapse [50]. In such cases, a 131I-MIBG therapy may be considered to control tumor growth and the patient’s symptoms. Notably, 131-MIBG is a form of therapy that depends on specific receptors. MIBG, which is an analog of guanethidine, shares structural similarity with norepinephrine and can bind to a receptor for this hormone [54]. Then, MIBG is transported to secretory granules or remains in the cytoplasm. In therapy, MIGB is conjugated with I-131, a radionuclide with meaningful β-particles emission. This emission accounts for cell damage due to their high mean energy and energy deposition [54]. A meta-analysis of 17 studies including 243 patients showed that stable disease (SD), and partial hormonal response may be obtained in over 50 % of patients with PGL and over 40 % with PCh [55]. In another study, on 131I-MIBG, Pryma et al. showed that it enables sustained blood pressure control and high 12-month tumor response rates (92% partial response (PR) or stable disease (SD); 68% PR or SD with a decrease in serum chromogranin levels) [56]. A study of long-term outcomes (median survival time from diagnosis was 11.5 years), conducted by Thorpe et al., reported that imaging, laboratory, and symptomatic response on treatment were obtained for both PCh and PGL [57]. Another potential option is a peptide receptor radionuclide therapy (PRRT), with lutetium-177 (177Lu)-labeled DOTA-Tyr3-octreotate (DOTATATE; oxodotreotide), yttrium-90 (90Y), or 177Lu-labeled somatostatin analogs (SSAs), which is intended for patients with metastases or patients disqualified for surgery [50,58]. Patients with metastatic PCh and PGL may receive chemotherapy (a combination of cyclophosphamide, vincristine, and dacarbazine is recommended) to alleviate the symptoms, although with poor effectiveness [45]. Another way of palliative treatment is an ablation of the metastatic tumors—patients with bone, chest-wall, and retroperitoneal metastases can undergo radiofrequency ablation or cryoablation, whereas patients with liver metastases should be treated with radiofrequency ablation or ethanol injection [48]. RT is also an option for symptomatic metastases regardless of the location of the tumor.

## 6. Future Directions in PGL Treatment

Treatment for malignant PCh and PGL tumors has still limited value. Thus, many clinical trials seeking a more targeted approach are being conducted [59]. Currently, 65 clinical trials are registered on ClinicalTrials.gov. In this section, we describe molecularly targeted agents and immunotherapy that could be relevant, as well as future therapeutic options for PCh and PGL management.

### 6.1. Molecular Targeted Agents

#### 6.1.1. Everolimus

Everolimus is a inhibitor of the mTOR pathway – a promising target in future therapies. To maximize its efficiency, the combination treatment was proposed, and subsequent studies on cell lines were conducted. Nölting et al. showed that everolimus in combination with lovastatin was significantly more effective (additive effect on inhibition) than everolimus alone [60]. In addition, in mouse PCh MPC and MTT cell lines, this combination inhibited EGFR and AKT signaling in the most prominent way [60]. Another study confirmed these results, revealing that treatment with everolimus and lovastatin significantly inhibits AKT and mTORC1/p70S6K signaling, without ERK upregulation [61]. Moreover, Fankhauser et al. sought to combine everolimus with BYL719 (PI3Ka inhibitor, alpelisib), earlier evaluated in breast cancer [62,63]. Although both inhibitors decreased cell viability in a relevant way, the combination was more effective (additive effect) [62].

The efficacy of mTOR inhibitors monotherapy was reported in the literature. Do-Youn et al. conducted a phase II study of everolimus in monotherapy in a heterogeneous group of patients with nonfunctioning NETs (*n* = 27) in which five had PCh, and two had PGL tumors. In this group of seven patients, five achieved SD, and two developed progressions (PDs). The median progression-free survival (PFS) was 3.8 months, and in four patients, radiological regression was observed [64]. Although the response was high in NETs, it was limited in both PCh and PGL [64]. Nevertheless, the data from such a small number of subjects are inconclusive and should be treated carefully. Furthermore, it is proved that, in both primary and metastatic PCh, the mTOR pathway is significantly dysregulated, with a preferential overactivation in head-and-neck PGL or in the group of patients with SDHX mutations (suggested by Oudijk et al.) [65,66,67,68,69,70]. However, inhibition of mTORC1 alone may not be enough because of compensatory activation of PIK3K/AKT and EGFR-related pathways, or RAS/RAF/ERK signaling [60,71,72,73,74,75].

#### 6.1.2. Axitinib

Axitinib, which is a VEGF receptor tyrosine kinase inhibitor previously investigated in renal-cell carcinoma, was assessed in metastatic, recurrent, or primary unresectable PCh or PGL tumors [76]. In the group of 12 patients, 5 achieved PR, 5 had SD, and 2 developed PD. The median of PFS was 7.7 (3.3–16.8) months.

#### 6.1.3. Cabozantinib

Cabozantinib is a multiple tyrosine kinases inhibitor of MET, RET, VEGFR2, and AXL [77]. One of the registered studies focuses on cabozantinib s-malate alone, whereas another study, on cabozantinib in combination with atezolizumab (CABATEN). Until now, the results of a phase I study on the use of cabozantinib in children and adolescents have been published. However, only one patient with PGL was included, achieving SD [77].

#### 6.1.4. Sunitinib and Sorafenib

Another promising drug is sunitinib, which is currently under investigation, with many studies supporting its role in gastrointestinal stromal tumors, advanced renal-cell carcinoma, and pancreatic NETs. Sunitinib is an orally administered drug and multitargeted inhibitor of receptor tyrosine kinases (RTKs) that mainly affects VEGFRs, PDGF-Rs, FLT3, RET, and mTOR signaling [78,79]. However, Saito et al. hypothesized that the effect of sunitinib is associated with angiogenesis inhibition as well as with direct antitumor effects [79].

Surprisingly, this drug-induced apoptosis of PC12 cell lines reduced the expression of antiapoptotic protein Bcl-2 activation of proapoptotic BAD and, crucially, led to an inhibition of the above-mentioned Akt and mTOR- related pathways, followed by a reduction in S6K1, the target of mTOR signaling [79]. Additionally, studying a xenograft model in mice, Denorme et al. confirmed the antiangiogenic activity of sunitinib and sorafenib. In addition, the numbers of apoptotic cells in vitro and in vivo in the treated tumors were higher, for both sunitinib and sorafenib, than those in control tumors, suggesting the direct effect on cells of these drugs [80].

Although results from basic models are promising, a phase II clinical trial (SNIPP) did not show considerable changes. In a group of 25 patients, the disease control rate was 83%, and the median PFS was 13.4 (range—5.3–24.6) months. Moreover, 3 patients achieved PR (all these patients had confirmed germline mutation), 16 achieved SD (>12 months), 4 developed PD, and the status of 2 patients is unknown [81].

#### 6.1.5. HIF Inhibitors

One of the promising but still weakly investigated drugs in PCh and PGL are hypoxia-inducible factor inhibitors (HIFs). Recent studies proved the potential feasibility and efficacy of HIF-2a inhibitors in the treatment of patients with clear-cell renal-cell carcinoma (RCC) (phase I, III, III of clinical trials) [18]. Due to similar molecular drivers present in RCC and both PCh and PGL, HIFs seem to be reasonable targets in these neoplasms [82].

Until now, available data indicate that, in both PGL and PCH, HIF-2a is overexpressed, while HIF1-a is not [83,84]. Therefore, currently, two clinical trials targeting HIF-2a are registered on ClinicaTrials.gov, and both have a “recruiting” status; the first is a phase II study of Belzutifan/MK-6482 monotherapy in advanced pheochromocytoma/paraganglioma, while the other is a phase I study of DFF332 in combination with everolimus, spartalizumab plus taminadenant in clear-cell renal-cell carcinoma and advanced hereditary PCh and PGL.

### 6.2. Anti-PD-1 Immunotherapy

Pembrolizumab, a checkpoint inhibitor of programmed cell death 1 (PD-1) used in the treatment of many types of cancer, was also investigated in relation to PCh and PGL. A phase 2 open-label trial on nine patients with these tumors showed that the nonprogression rate (NPR) assessed within 27 weeks was 43%, objective response rate (ORR) was 0%, and clinical benefit rate (CBR) was 75% [85]. Jimenez et al. revealed that, in a population of 11 patients treated with pembrolizumab, 40% achieved NPR (at 27 weeks), 9% ORR, and 73% CBR. PFS was 5.7 months (range: 4.37–unreached) [86].

### 6.3. Cancer Vaccines

Interestingly, one of the trials (1/2 phase) will investigate a novel therapeutic vaccine (EO2401) in combination with nivolumab, a PD-1 inhibitor [87]. Currently, some studies seek to use vaccines against PCh and PGL. For example, the attenuated Newcastle Disease Virus vaccine showed a cytotoxic effect on the PC12 rat pheochromocytoma cell line in two independent studies [88,89]. Another study focused on chromogranin A-based vaccine. In vitro analyses showed that, in vaccinated mice, a large increase in CgA-specific cytotoxic cells was noted. PCh tumors (exogenously applied) were significantly infiltrated by CD8+ cells. The lysis of the tumor and a reduction in its size were confirmed in an experimental model of PCh [90].

Although numerous promising drugs have been investigated, the future of PCh and PGL treatment remains uncertain. Clinical trials related to these tumors are hard to execute due to relatively small groups of patients diagnosed with progressive PCh and PGL and difficulties with recruitment. Multicenter and international collaborations are needed to make significant progress in the treatment of PCh and PGL tumors.

## 7. Discussion

Our case report emphasizes the role of metoxycatecholamines in the observation of patients after an incidence of PCh and PGL. Despite the relatively low cost and noninvasiveness of metoxycatecholamines measurement, it enables early detection of local and distant relapse or a second primary tumor in another location. Taking mentioned data into consideration, metoxycatechelomines can be treated as useful parameters that allow an appropriate level of oncological vigilance to be maintained and should be measured up to even 10 years after diagnosis at follow-up visits [36]. In our center, we usually recommend the measurement of metoxycatecholamines every 3 months for hormonally active tumors, whereas for inactive tumors, the follow-up is not conducted. In the case of PGL tumors, fast diagnosis may be crucial for survival. Metoxycatecholamines are also extremely useful in monitoring the course of the disease and the efficiency of the treatment [91,92,93].

As we also described, in our case study, the abdomen, chest, and head-and-neck CT, as well as somatostatin receptor scintigraphy, did not reveal the presence of PGL early after biochemical relapse. The decision of conducting CT as a first-line method is based on guidelines and the evidence that head-and-neck PGL cause catecholamine hypersecretion significantly less frequently than PGL tumor located in other parts of the body [93]. In addition, the first PGL tumor of our patient was PCh, which made recurrence in the abdomen region more probable. We started to consider different imaging methods to provide a faster diagnosis of a tumor in an unknown location. We finally chose [68] Ga-DODATE PET and subsequently MRI with intravenous contrast, which, under these circumstances (negative CT and SRS), seemed to offer the highest sensitivity. Both techniques confirmed considerable metabolically active PGL in the neck region (infiltrating the sublingual nerve, closing the internal jugular vein, and reaching the left internal carotid artery canal). Contrary to the guidelines [32,36], we performed first [68] Ga-DODATE PET, before MRI imaging. The decision was taken with a consideration of patients’ exhaustion triggered by prolonged diagnostic procedures associated with the peculiarity of the case and the unfavorable COVID-19 outbreak period. PET provided rapid information about the location of the tumor, which was subsequently confirmed and evaluated by MRI. We started to consider whether it is advisable to perform PET before CT or MRI, specifically in the case of PGL tumors in unknown locations or with suspected metastatic characteristics. Han et al. conducted a meta-analysis of 13 studies, which proved that pooled detection rate of PET is approximately 93%, indicating its high diagnostic value, comparable to or even more relevant than other imaging methods [94]. Following the guidelines [32,36], in our case, we had to perform three imaging steps. First, CT had to be performed to confirm or reject the diagnosis of the most probable PGL tumor located in the abdomen or chest. Second, after exclusion of the first clinical hypothesis, MRI had to be performed to find potential PGL located in the head and neck region. Then, in the end, PET had to be performed, as a tumor is known for its high probability of metastasis. Performing PET before CT and MRI may be more beneficial, enabling the elimination of unnecessary diagnostic steps, which may reduce costs and risk of exposure for patients. It seems to be valuable, especially in the case of uncooperative patients. It is often observed in daily practice that prolonged diagnosis prompts patients to therapy refusal. In our case, the patient was not determined to complete the diagnostic process. In combination with limited access to imaging during the COVID-19 pandemic, it prolonged the period when the patient received the first line of treatment up to several months.

Our case supports an observation that the COVID-19 pandemic has influenced the health care system and has disrupted the management of various diseases [95,96]. The patient was afraid of visits to the hospital due to the risk of COVID-19 infection, which was the main reason for the interruption of medical procedures. Lazzerini et al. also observed that effect during the pandemic in Italy [97]. Additionally, some studies report that waiting time significantly influences patients’ satisfaction and engagement with therapy and may lead to more intensive pain or anxiety [98,99,100,101,102]. Hence, the need to limit the number of necessary procedures, which may be beneficial to final patient outcomes. It may also significantly decrease costs, without a lack of related decrease in specificity and sensitivity. Moreover, it may also reduce the potential COVID-19 exposure in medical centers during these procedures. According to the Working Group on Endocrine Hypertension of the European Society of Hypertension, we have two available options, i.e., 18F-FDOPA positron emission tomography (18-FD) and 68Ga-DOTA-somatostatin analog (Ga-68). We finally used the Ga-68, which provided us with a fast diagnosis. Our approach seems to be relevant to the recently published umbrella meta-analysis conducted by Treglia et al. That study showed that, in the case of PCh and PGL, the diagnostic value (measured by lesion-based pool detection rate) of SSA is higher than that of 18-FD or 18F-FDG (respectively, 93%, 80%, 74%) [103].

Genetics seems to be crucial in PCh and PGL tumor development. As we reported in Section 3. *Genetic Background*, genetics may influence the diagnosis, treatment, and prognosis of patients. A molecularly targeted approach may be the future of diagnosis and treatment of PCh and PGL. Although current knowledge of a clinical outlook associated with specific mutations is still insufficient, we can make effort to adjust our diagnosis and treatment approaches to a specific gene, especially if clinical symptoms strongly suggest one of the mutations or syndromes. In order to make gene-based decisions easier, we summarized the data available in the literature and associated them with selected mutations, indicating also missing parts in the research. Although there are still some missing parts in the literature, considerable progress has been made since the first study revealed RET proto-oncogene as the risk factor for PGL in 1993 [93,104]. There is a need for more studies that will fill the gaps and make the management of PCh and PGL tumors more personalized and effective.

## Figures and Tables

**Table 1:** Changes in clinical findings with time.

Metoxycatecholamines Tested	Levels of Metoxycatecholamines in 24 h Urine Test		
	January 2020 (µg/Ml)	March 2020 (µg/24 h)	August 2021 (µg/24 h)
Metanephrine	550	88.9	65.1
3-Methoxytyramine	1047	356.5	224.8
Normetanephrine	6902	1858.2	1180.5

**Table 2:** Clinical data according to a mutation. Summarized data available in the literature. Non-specific (NS) [15,16,17,18,19,20,21,22,23,24,25,26,27,28,29,30,31].

Cluster	Mutated Gene	Frequency of Mutation	Mechanism	Hormonal Activity	Syndrome Name	Preferred Image Diagnostic	Main Localization	Treatment
Pseudohypoxic cluster	VHL	7%	Accumulation of HIF-2 α	NE, NMT	VHL	18F-FDOPA	Abdomen	α or β receptor inhibitors, surgery
	SDHD	9%	Accumulation of fumarate and succinate	NE, NMT, DA, 3-MT	PGL 1	1st choice: 68Ga-somatostatin analog PET/CT 2nd: F-FDG avidity PET/CT	Head and neck	α or β receptor inhibitors, surgery chemotherapy (cyclophosphamide, vincristine, acarbazine, temozolomide)
	SDHAF2	<1%			PGL 2	1st choice: 68Ga-somatostatin analog 2nd: F-FDG avidity	Chest, carotid body	
	SDHC	0–6.6%			PGL 3	1st choice: 68Ga-somatostatin analog 2nd: F-FDG avidity	Chest, head and neck, carotid body	
	SDHB	10%			PGL 4	1st choice: 68Ga-somatostatin analog 2nd: F-FDG avidity	Chest, abdomen	
	SDHA	3% of sporadic PPGL			PGL 5	1st choice: 68Ga-somatostatin analog 2nd: F-FDG avidity	Head and neck, abdomen	
	EGLN1/2/3	2 patients	No regulation of the stability of HIF-α by PDH-1,-2,-3	NE, NMT	–	18F-FDOPA PET/CT	Abdomen	α or β receptor inhibitors, surgery
	HIF2A	2 patients	Dysregulation of adaptation to hypoxia	NE, NMT	Pacak-Zhuang syndrome	Avid F-FDOPA and F-FDG uptake 18F-FDOPA PET/CT	Abdomen	α or β receptor inhibitors, surgery
	IDH	1 patient	Accumulation of 2-hydroxy, glutarate	NA	NA	NA	NA	α or β receptor inhibitors, surgery
	MDH2	5 patients	Tumor suppression gene mutations	NE, NMT	NA	NA	Chest, abdomen	α or β receptor inhibitors, surgery
Kinase receptor signaling	RET	6%	Activation of Ras/MAPK and PI2K/AKT signaling	NE, NMT, EPI, MT, N-methyltransferase	MEN-2	18F-FDOPA	Adrenal medulla	α or β receptor inhibitors, surgery
	FH	NA	Accumulation of fumarate, succinate	NE, NMT	NA	68Ga-DOTATATE PET/CT	NA	α or β receptor inhibitors, surgery
	NF1	5–7%	mTOR signaling activation	adrenergic phenotype	NF type 1	18F-FDOPA PET-CT	Adrenal	α or β receptor inhibitors, surgery
	MAX	1.1%	Myc signaling activation	NE, NMT	Familial PHEO	1st choice: 18F-FDOPA PET/CT	Abdomen	α or β receptor inhibitors, surgery
	TMEM127	2%	mTOR signaling activation	NMT, MT	Familial PHEO	1st choice: 18F-FDOPA PET/CT	Abdomen	α or β receptor inhibitors, surgery
	H-RAS	5.2% (small group of patients)	Ras mutation	Adrenal, adrenergic phenotype	NA	1st choice: 18F-FDOPA PET/CT	Adrenal	α or β receptor inhibitors, surgery
	K-RAS	NA			NA	1st choice: 18F-FDOPA PET/CT	Adrenal	α or β receptor inhibitors, surgery
	ATRX	1 patient	Loss of function of ATRX	Noradrenergic phenotype	NA	NA	Adrenal	α or β receptor inhibitors, surgery
Wnt signaling cluster	CSDE1	4 patients	Loss of function of CSDE1	Adrenal, adrenergic phenotype	NA	NA	Adrenal	α or β receptor inhibitors, surgery
	MAML3	NA	Increased Wnt and Hedgehog signaling	NE, NMT, EPI, MT	NA	NA	Adrenal	α or β receptor inhibitors, surgery

Abbreviations: NE—norepinephrine; NMT—normetanephrine; EPI—epinephrine; MT—metanephrine; DA—dopamine; 3-MT—methoxytyramine, NA—not available; VHL—von Hippel–Lindau.

**Table 3:** Biochemical diagnosis of PGL tumors; HPLC—high-performance liquid chromatography.

Recommended Material and Method	Catecholamine	Example of a Reference Norm
24 h urine test, HPLC	Noradrenaline	15–80 µg/24 h
24 h urine test, HPLC	Adrenaline	0–20 µg/24 h
Urine, free metanephrines, spectrophotometrically	Metoxyadrenaline	0–12 µg/24 h
Urine, spectrophotometrically	Vanillinmandelic acid	0–7.9 mg/24 h
Plasma, HPLC	Noradrenaline	80–498 pg/mL
Plasma, HPLC	Adrenaline	4–83 pg/mL
